# Interactions of the EphA2 Kinase Domain with PIPs in Membranes: Implications for Receptor Function

**DOI:** 10.1016/j.str.2018.05.003

**Published:** 2018-07-03

**Authors:** Matthieu Chavent, Dimple Karia, Antreas C. Kalli, Jan Domański, Anna L. Duncan, George Hedger, Phillip J. Stansfeld, Elena Seiradake, E. Yvonne Jones, Mark S.P. Sansom

**Affiliations:** 1 Department of Biochemistry, University of Oxford, South Parks Road, Oxford OX1 3QU, UK; 2 Division of Structural Biology, Wellcome Centre for Human Genetics, University of Oxford, Roosevelt Drive, Oxford OX3 7BN, UK; 3 Institut de Pharmacologie et de Biologie Structurale IPBS, Université de Toulouse, CNRS, UPS, Toulouse, France; 4 Leeds Institute of Cancer and Pathology, St James's University Hospital, University of Leeds, Leeds, UK

**Keywords:** EphA2 kinase, cell membrane, juxtamembrane, phosphatidylinositol phosphates, molecular dynamics, multi-scale simulations

## Abstract

EphA2 is a member of the receptor tyrosine kinase family. Interactions of the cytoplasmic region of EphA2 with the cell membrane are functionally important and yet remain incompletely characterized. Molecular dynamics simulations combined with biochemical studies reveal the interactions of the transmembrane, juxtamembrane (JM), and kinase domains with the membrane. We describe how the kinase domain is oriented relative to the membrane and how the JM region can modulate this interaction. We highlight the role of phosphatidylinositol phosphates (PIPs) in mediating the interaction of the kinase domain with the membrane and, conversely, how positively charged patches at the kinase surface and in the JM region induce the formation of nanoclusters of PIP molecules in the membrane. Integration of these results with those from previous studies enable computational reconstitution of a near complete EphA2 receptor within a membrane, suggesting a role for receptor-lipid interactions in modulation of EphA2.

## Highlights

• Molecular simulations unravel how EphA2 kinase interacts with PIP_2_ in membranes
• The EphA2 juxtamembrane (JM) domain interacts with the kinase domain and membrane
• The JM and kinase domains drive formation of PIP_2_ nanoclusters in the membrane
• An integrative model supports *trans* autophosphorylation within clustered EphA2s

## Graphical Abstract

**Figure:**
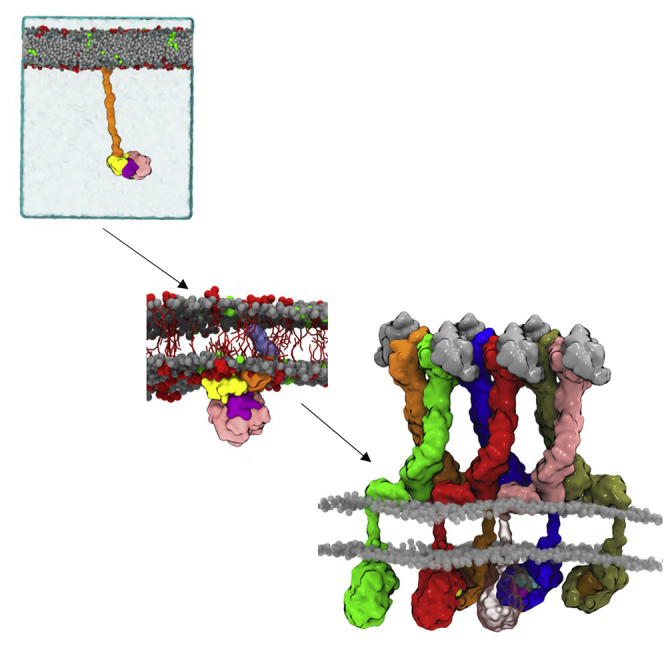

## Teaser

Chavent et al. investigate interactions of the EphA2 receptor tyrosine kinase with a membrane. Phosphatidylinositol phosphates (PIPs) mediate interaction of the kinase domain with the membrane, while kinase and juxtamembrane domains induce formation of nanoclusters of PIP molecules. These results enable computational reconstitution of a near complete EphA2 receptor model.

## Introduction

The ephrin receptors (Ephs) are key members of the family of receptor tyrosine kinases (RTKs). They have critical roles in developmental processes and have been implicated in a number of cancers (Herbert and Stainier, 2011, Lai and Ip, 2009, Pasquale, 2010). Ephs are grouped into two classes, A and B. Class A Ephs bind preferentially to ephrinA ligands, which are membrane-tethered through a glycosylphosphatidylinositol (GPI) anchor. Class B Ephs preferentially bind ephrinBs, which are attached to the membrane via a transmembrane (TM) helix (Kullander and Klein, 2002). All Eph receptors share a common domain architecture, whereby the N-terminal ectodomain is made up of a ligand binding domain (LBD), which interacts with ephrin ligands, a Sushi domain, an epidermal growth factor (EGF)-like domain, and two fibronectin type III domains (FN1 and FN2). The intracellular region contains a tyrosine kinase domain, a sterile alpha-motif (SAM) domain, and in some receptor species a PDZ binding motif. A single TM helix (Bocharov et al., 2010, Chavent et al., 2014), followed by an extended (ca. 40 residues) juxtamembrane (JM) linker rich in basic residues in its first half, which connects the ectodomain and the intracellular regions. Recent crystal structures of the ectodomains of two Eph receptors, EphA2 and EphA4, in complex with and without ephrin ligands, have revealed that ligand-induced EphA clustering is driven by a conserved Sushi-Sushi interaction with LBD-LBD interactions providing additional, receptor-specific, contributions (Himanen et al., 2010, Seiradake et al., 2010, Seiradake et al., 2013, Xu et al., 2013). Recent combined simulation and biochemical studies (Chavent et al., 2016) suggest the ectodomain may be oriented relative to the cell membrane via FN2 domain interactions with lipids in the extracellular leaflet.

There have been numerous structural studies of the isolated domains (ectodomain, TM domain, and kinase domain) of RTKs, especially of the EGF receptor (EGFR) and related receptors (Bessman et al., 2014). However, understanding how to put these structures back together in a model of the functional receptor in a membrane remains challenging (Arkhipov et al., 2013). Furthermore, it is important to include considerations of how membrane lipids, especially glycolipids (Coskun et al., 2011) and phosphatidylinositol phosphates (PIPs) (Michailidis et al., 2011) interact with these receptors within cell membranes.

Molecular dynamics (MD) simulations enable the study of the dynamic interactions of lipids with receptors and related membrane proteins (Hedger and Sansom, 2016). In addition to providing structural and biophysical information on the interactions of lipids with the TM domains of membrane proteins, they may be used to study the interactions of peripheral proteins and/or domains with the surfaces of complex cellular membranes (Venken et al., 2017, Yamamoto et al., 2016). Thus, simulation studies have been used to explore possible interactions of, e.g., the TM domains of the EphA1 receptor (Chavent et al., 2014) and of the ectodomain of the EphA2 receptor with the lipid bilayer (Chavent et al., 2016). There have also been a number of simulation studies of the related EGFR (e.g., Arkhipov et al., 2013, Endres et al., 2013, Kastner et al., 2009, Kaszuba et al., 2015, Lelimousin et al., 2016).

**Figure 1:**
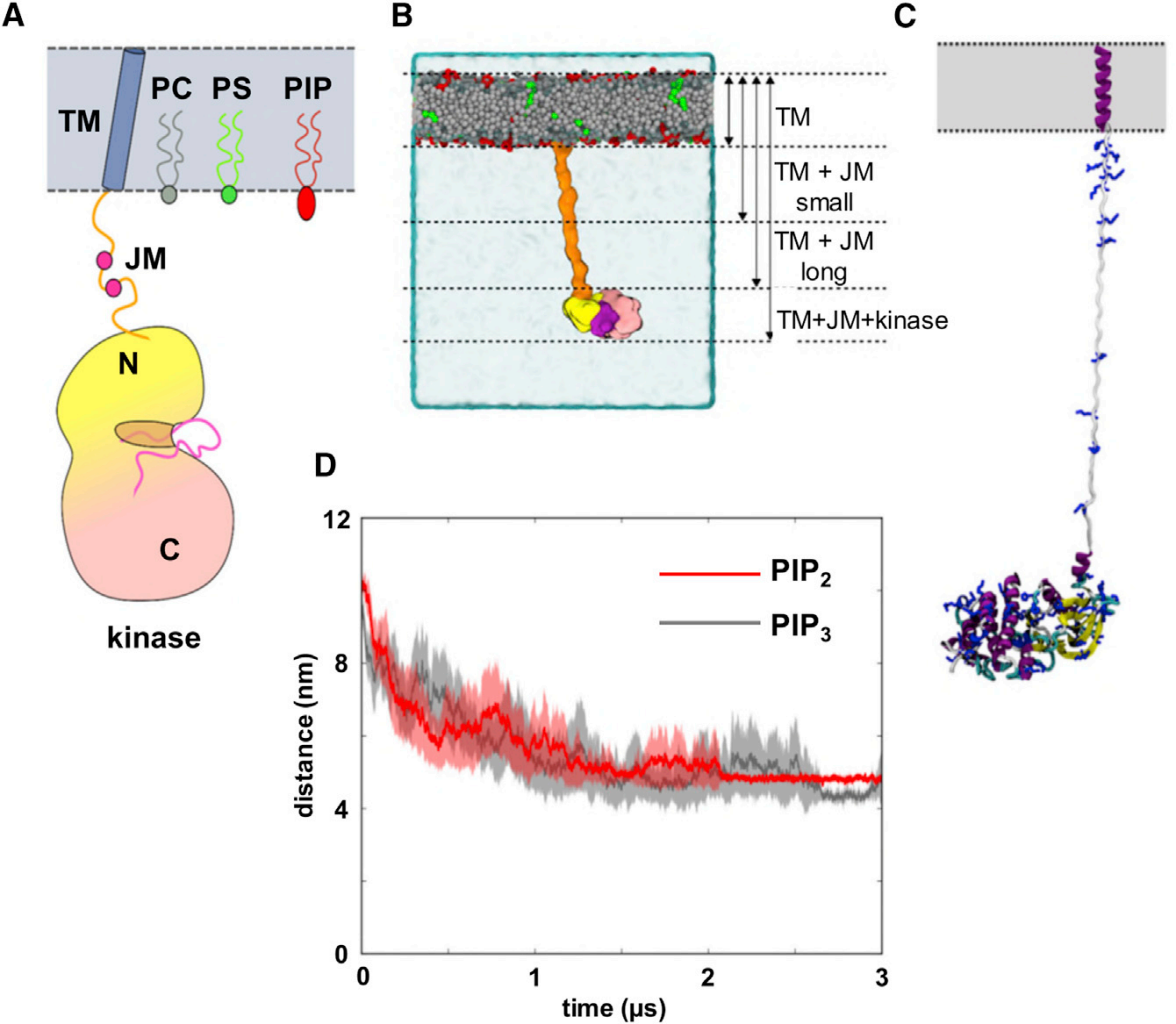
The Juxtamembrane and Kinase Domains of EphA2 at the Membrane (A) Schematic representation of the first (i.e., N-terminal part) of the cytosolic domains of EphA2, showing the transmembrane (TM; blue) helix domain followed by a juxtamembrane (JM; orange) segment, and the kinase domain (N-terminal lobe in yellow, the C-terminal lobe in pink, and the activation loop in pink). The lipid bilayer is shown in gray with the three classes of lipid included in our simulations: phosphatidylcholine (PC; gray), phosphatidyl serine (PS; green) and phosphatidyl inositol phosphates (PIP; in red). The two mauve circles in the JM region depict the conserved Tyr motif (Y588 and Y594 for EphA2) that can be phosphorylated. (B) Coarse-grained model of the EphA2 JM + kinase domains tethered at a membrane by the TM domain (hidden by lipids). In the starting configuration, we modeled the JM segment as extended but flexible. Upon simulation the JM domain collapsed onto the bilayer surface drawing the kinase domain toward the membrane (see Video S1). Simulations (see Table 1 for details) were performed for systems with just the TM domain with one or two extensions of the JM domain in addition to the TM + JM + kinase system depicted. (C) Starting tethered model in atomistic resolution showing the positively charged residues in the JM and kinase domains. (D) Evolution of the average distance between the centers of mass of membrane and the untethered kinase domain for simulations (see Table 1) in which the bilayer contained PIP_2_ (red) or PIP_3_ (gray). A distance of 4–5 nm indicates a stable interaction between the kinase and the membrane. In each case the bold line shows mean distance for each set of ten simulations and the transparent background the SEM. See also Figure S1 for further details of these simulations.

In contrast to the ectodomain, the molecular organization of the JM and cytoplasmic region of the EphA2 receptor and its interactions with the cell membrane remain poorly understood. Studies of the related EGFR (Arkhipov et al., 2013, Endres et al., 2013, Hedger et al., 2015, Hedger et al., 2016a) suggest that interactions of the JM and tyrosine kinase domains with the intracellular face of the membrane may play a key role in the mechanism(s) of receptor activation. In the current study, we have used coarse-grained (CG) molecular simulations alongside a biochemical assay to characterize the interactions of the immediate cytosolic part of the EphA2 receptor (i.e., the JM + kinase domains) with the lipids of the surrounding membrane. We have characterized how the JM + kinase domains interact with anionic lipids (especially the phosphatidylinositol phosphates PIP_2_ and PIP_3_; Figure 1A) within models of the cell membranes. By combining these results with our previous study of the ectodomain interactions with a membrane, we are able to propose a near full-length model of the EphA2 receptor within a membrane, which can be used to suggest a role for receptor-lipid interactions in EphA2 activation.

## Results and Discussion

### Interaction of the EphA2 Kinase Domain with Model Cell Membranes

Inspection of the location of basic residues both within the JM region (predicted to be largely unstructured; Figure 1C) and on the surface of the kinase domain suggest that the components of the JM + kinase region are likely to interact with anionic lipids at the cytoplasmic surface of the cell membrane. Furthermore, previous studies had suggested PIP_2_ interactions with the proximal juxtamembrane region of a TM + JM fragment of EphA2 (Hedger et al., 2015). We therefore wished to explore the interactions of the kinase domain in isolation with membranes and their (anionic) lipids, and subsequently of the kinase domain tethered to the TM domain by the JM region with membranes and their (anionic) lipids (Figure 1B).

To explore the intrinsic propensity of the kinase domain to bind to anionic lipids in a bilayer, we launched an ensemble of ten simulations of the isolated kinase domain, which was initially positioned distant from the membrane surface, such that the center of mass distance between the protein and lipid bilayer was ca. 10 nm (see Figure S1). Starting in the aqueous phase, the protein diffused until it reached the membrane surface where interaction with lipid molecules took place. It should be noted that this encounter with the membrane could occur on either side (as a consequence of periodic boundary conditions; see STAR Methods). Measuring the encounter process and the subsequent interactions of the protein with the membrane surface (both averaged across the ensemble) enables us to assess the relative strength of the interaction of the kinase domain with bilayers of different lipid compositions. The resultant interaction was shown to be sensitive to the nature of the anionic lipid species present in the bilayer. Thus, only very transient interactions were formed between the kinase and a phosphatidylcholine (PC):phosphatidylserine (PS) membrane regardless of whether 30% or 40% PS was present in the bilayer. In contrast, if either PIP_2_ (5 mol%) or PIP_3_ (2 mol%) was included in the bilayer, sustained interactions were formed (see Figures 1D and S1; Video S1). These interactions were generally formed within the first 1 μs and lasted for >2 μs. Inspection of individual simulations within each ensemble (Figure S1) reveals a couple of cases for PIP_3_, where interaction of the kinase with the bilayer was followed by subsequent (albeit transient) dissociation. Note that lipid ratios of PC:PS 70:30, PC:PS:PIP_2_ 90:5:5, and of PC:PS:PIP_3_ 83:15:2 were used to approximate the lipid composition of a mammalian cell membrane (van Meer et al., 2008), while maintaining a constant net charge on the surface of the model membranes. Based on this set of simulations and the likely limitations of the CG simulation approach employed (see below for a more detailed discussion) it is difficult to comment with any degree of certainty on possible selectivity for PIP_2_ versus PIP_3_.

Video S1. Diffusion of the Untethered Kinase Domain toward a Model Membrane Containing PC, PS, and PIP_2_ Lipids, Related to Figure 1 The colored bar at the bottom shows the distance in between the center of masses of the kinase and the membrane from white (>12 nm) to black (∼4 nm). For further information see Figure S1.

**Figure 2:**
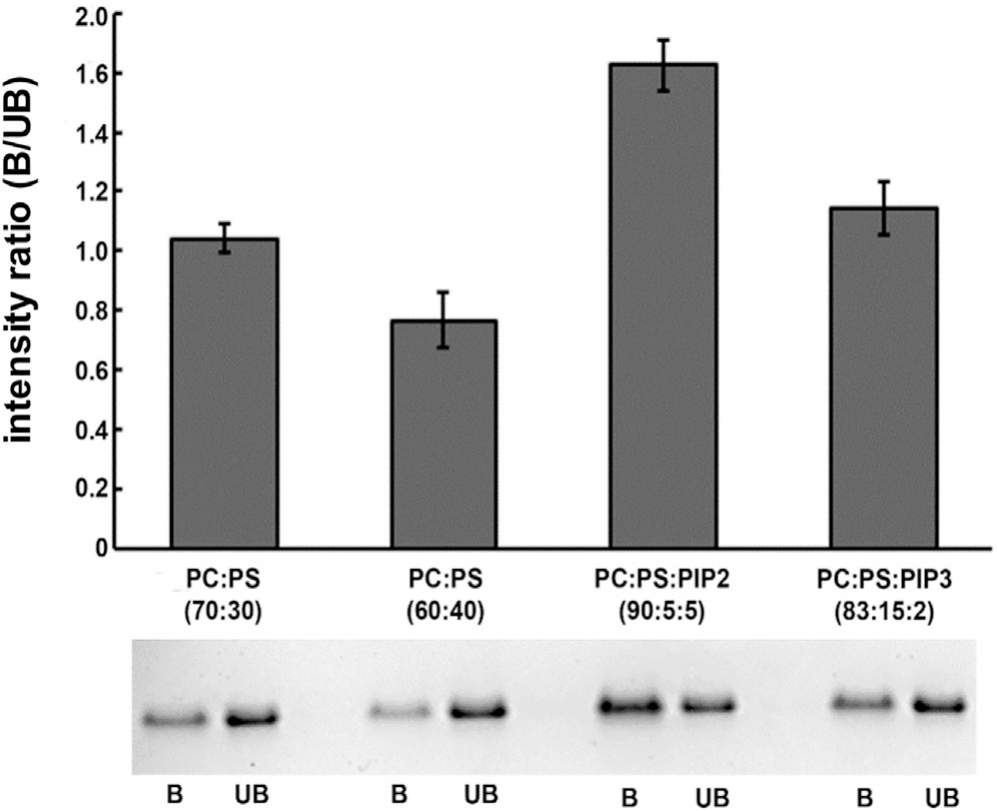
Liposome Pull-Down Experiment Showing the Interaction of Kinase Domain with PC:PS, PC:PS:PIP_2_, and PC:PS:PIP_3_ Bilayers Intensities of the bound fractions for each liposome composition were measured on SDS-PAGE. Averages of the ratio of intensities (bound:unbound) and SEM (error bars shown) were then calculated for all (n = 5) experiments. B and UB represent the bound and unbound fractions, respectively.

To evaluate our computational results, we performed liposome pull-down assays of the kinase domain using different lipid compositions (see Figure 2). These experiments indicate that the presence of anionic lipid alone (i.e., PS at 30% or 40%) does not lead to an appreciable fraction of the protein in a liposome-bound state. In contrast, in the presence of 5% PIP_2_ (which may be an underestimate of the localized concentration under physiological conditions) there was a substantial bound fraction of protein. This does not seem to be the case in the presence of 2% PIP_3_. Although the kinase domain when bound to liposome may include autophosphorylated and/or multimerized species, these experiments do demonstrate that the EphA2 kinase domain has an intrinsic ability to bind to PIP_2_-containing membranes consistent with that observed in the simulations.

Taken together, this combination of MD simulations and liposome pull-down assays indicates that the isolated EphA2 kinase domain has an intrinsic propensity to bind to PIP_2_ molecules within a phospholipid bilayer. The simulations were therefore extended to explore the nature of such interactions in the context of a kinase domain “tethered” to a membrane by a JM region linking it to a TM helix.

### EphA2 Kinase Has Two Modes of Interaction with PIP-Containing Membranes

**Figure 3:**
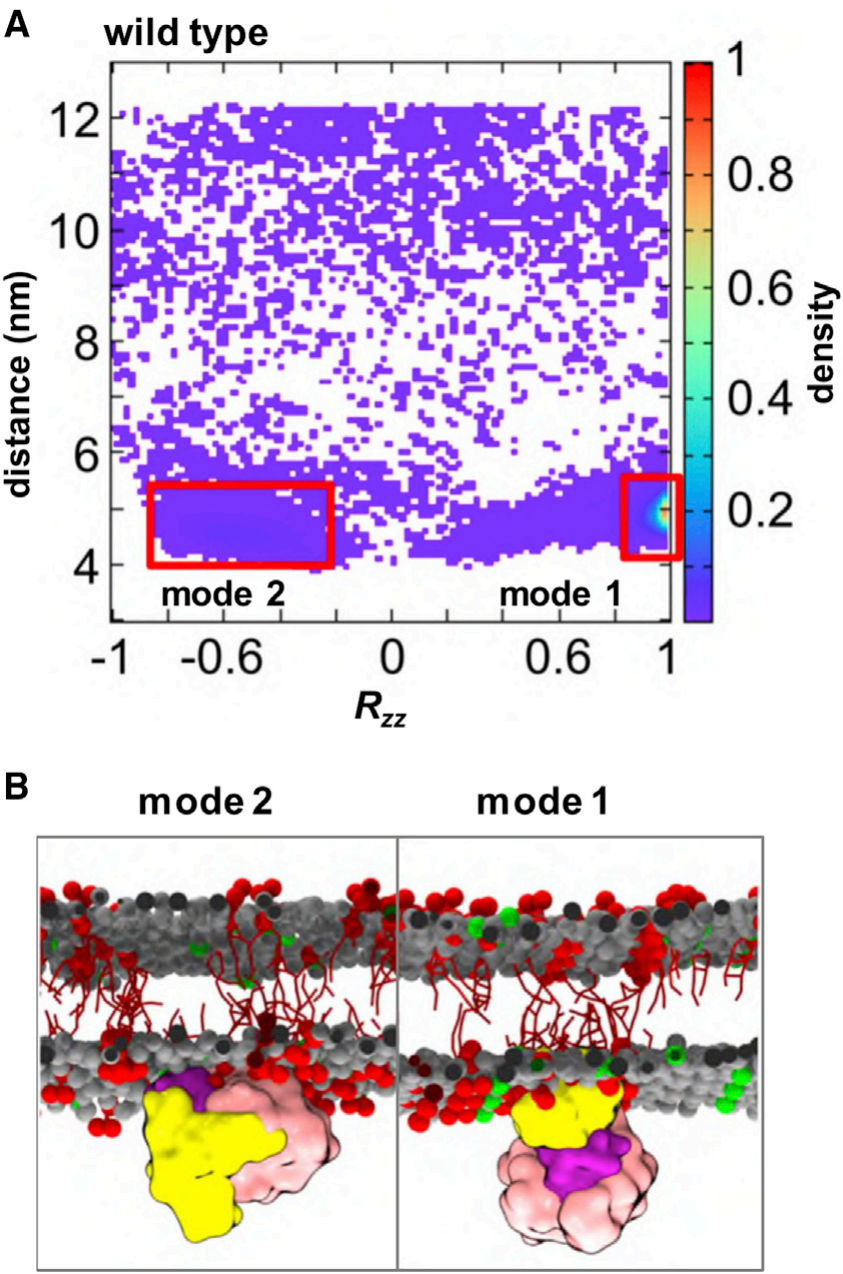
Interaction of the Untethered EphA2 Kinase Domain with a PIP_2_-Containing Membrane (A) Position and orientation of the (wild-type) kinase domain throughout the simulation (derived from ten simulations, see Table 1) displayed as a normalized density map showing the domain-bilayer centers-of-mass separation *d* and *R*_*zz*_ component of the rotation matrix. The two main modes of interaction of the kinase with the bilayer are highlighted via red boxes. (B) Examples of the two main modes of interaction of the kinase domain with the membrane. See also Figure S2 for an example of the transition from mode 2 to mode 1 and orientations of the mutants.

**Figure 4:**
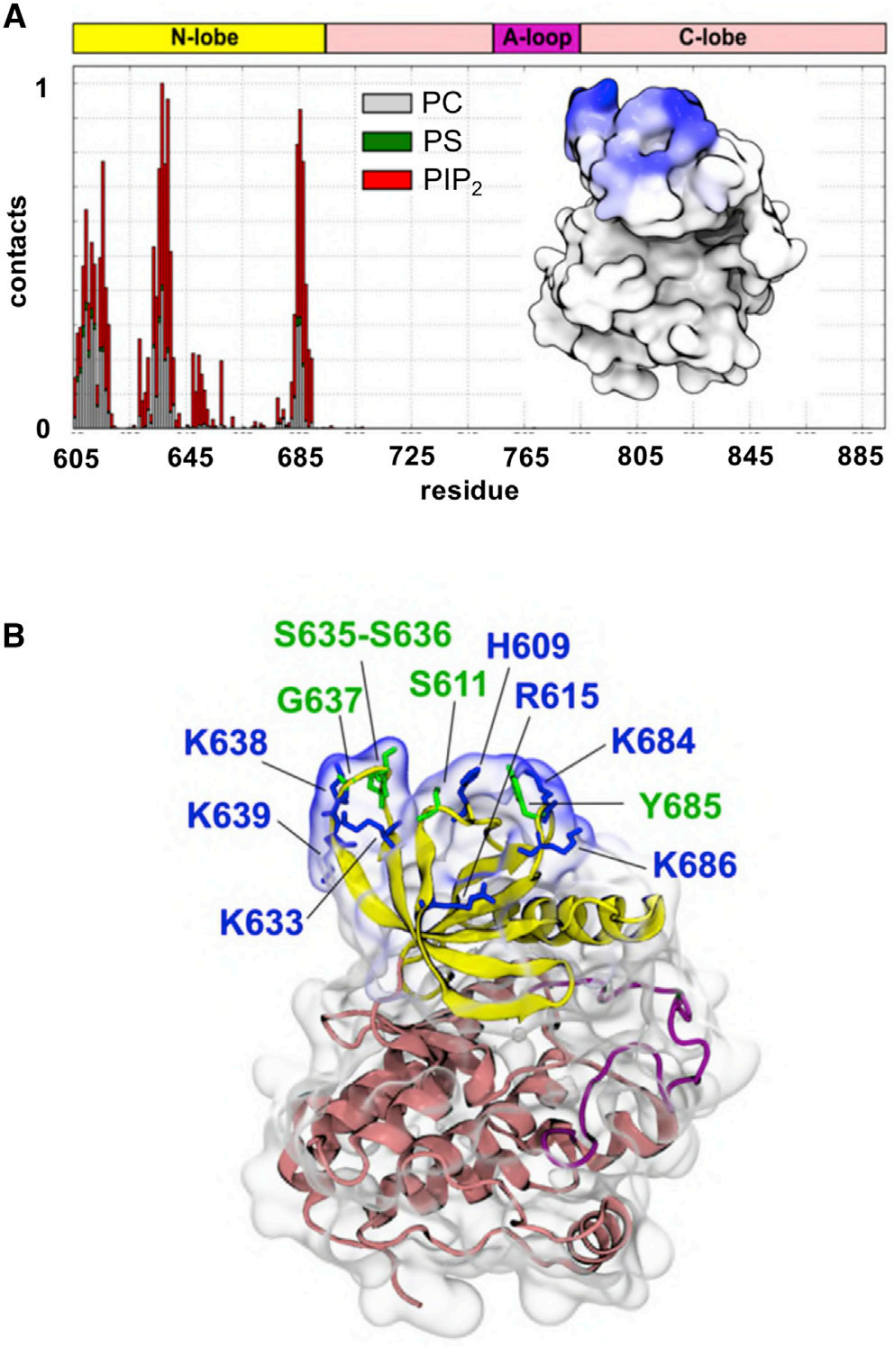
Kinase Residues Interacting with PIP_2_ Molecules (A) Normalized frequency of contacts (defined as the relative number of interacting particles within a 0.8 nm cutoff distance of the PIP_2_ head group) between the kinase domain and the PIP_2_-containing membrane for the mode 1 interaction (see Figure 3). Red depicts interactions with PIP_2_ molecules, green with PS, and gray with PC. The interacting residues are all located in the N-terminal lobe of the kinase, as illustrated in the inset, which depicts the contacts highlighted at the protein surface by a white to blue gradient. (B) Residues (basic in blue, others in green) interacting with lipid molecules in the membrane for the mode 1 interaction. The results of comparable analysis for the mode 2 interaction are presented in Figure S3.

To further understand how PIP molecules can mediate the interaction of the kinase domain with model cell membranes, we performed an orientational analysis of our MD simulations. This approach has proved to be useful in characterizing PIP-dependent interactions with, e.g., pleckstrin homology (PH) domains (Yamamoto et al., 2016). Analysis of the orientation of the kinase domain when approaching and when bound to the PIP_2_-containing membrane suggests that there are two main modes of interaction (Figure 3). The predominant binding mode (mode 1 in Figure 3) involves the N-terminal lobe of the kinase domain. In this interaction mode, the activation loop of the kinase is accessible to phosphorylation. In the secondary mode (mode 2 in Figure 3), the interaction with the bilayer involves both the N- and C-terminal lobes of the kinase and thus the activation loop is masked. These binding modes were also observed in the simulations with PIP_3_-containing membranes (see Figure S2). Averaging across the two ensembles of simulations showed a majority of interactions via mode 1 (15/20 simulations), while mode 2 was present in 5/20 simulations. Thus, mode 1 seems to be the preferred mode of interaction with the membrane. This may be also highlighted by the fact that it is possible to see the transition from mode 2 to mode 1 during the course of the simulation (see Figure S2). Both modes of interaction were mainly driven by positively charged and polar residues on the surface of the kinase (see Figures 4 and S3), but the key difference thus lies in the accessibility of the activation loop of the kinase. We then refined these two modes of interaction by using them as the starting points for short (100 ns) atomistic simulations. The protein interactions with lipids in these atomistic simulations remained in agreement with the CG simulations (see Figure S6).

To examine the importance of these interactions on the formation of the EphA2 kinase/membrane complex we performed *in silico* mutations targeting different parts of the protein surface forming the mode 1 protein-membrane interface (PMI). This PMI is formed by two patches on the surface of the kinase: residues H609, R615, and K617 forming patch 1 (P1); K629, K633, K638, and K639 forming patch 2 (P2). The residues of P1, P2, and of P1+P2 were mutated to aspartic acid. For each of the mutants (P1, P2, and P1+P2) we performed 10 simulations of kinase association with PIP_2_-containing membranes. Although these (*in silico*) mutations did not completely prevent interactions with the membrane, they switched the interaction to predominantly mode 2 (see Figure S2), i.e., the mode in which the activation loop is masked by the membrane.

### The JM Region Modulates Interactions of the Kinase Domain with the Membrane

**Figure 5:**
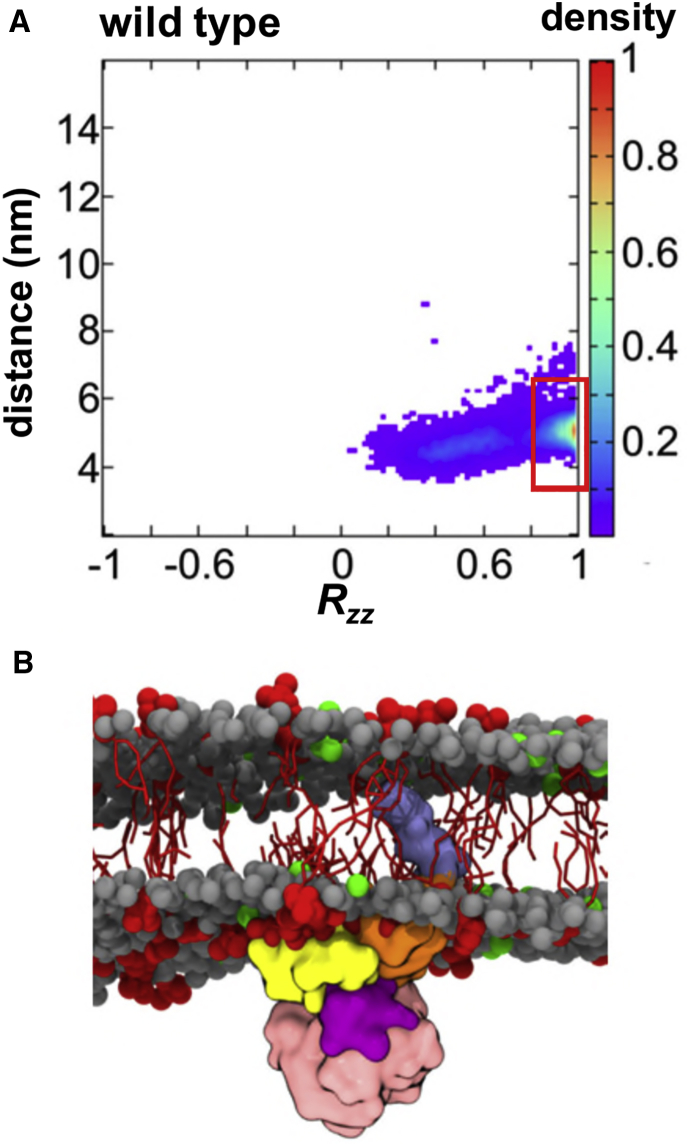
Interaction of the Tethered EphA2 Kinase Domain with a PIP_2_-Containing Membrane (A) Position and orientation of the tethered wild-type kinase domain (derived from data acquired across the three simulations; see Table 1 for details) displayed as a normalized density map showing the domain-bilayer centers-of-mass separation *d* and *zz* components of the rotation matrix *R*_*zz*_. The main modes of interaction of the kinase with the bilayer is highlighted via a red box. (B) Examples of the main mode (mode 1) of interaction of the tethered kinase domain with the membrane. See also Figure S4 for comparable analysis of the *in silico* mutant simulations.

**Figure 6:**
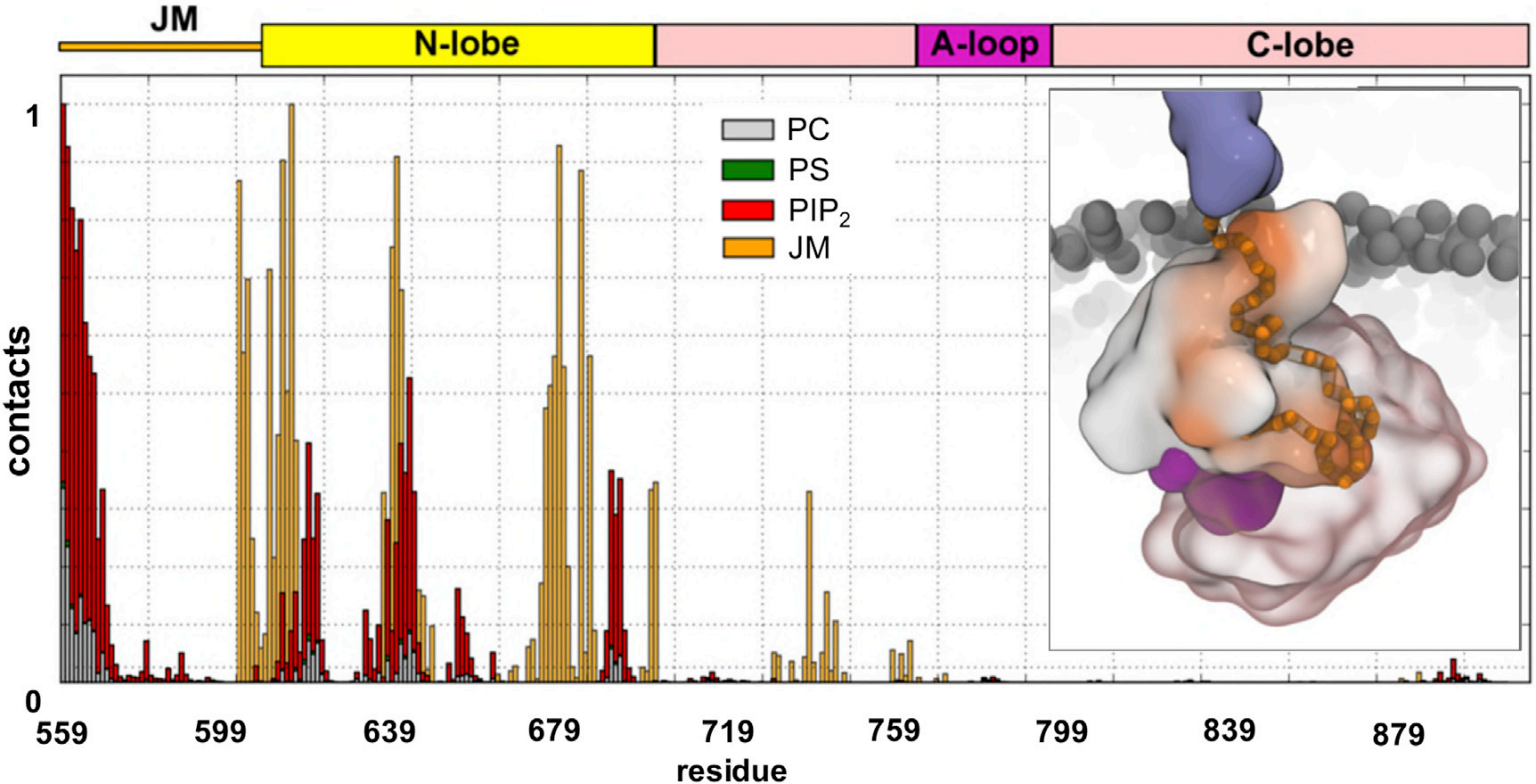
Kinase Residues Interacting with PIP_2_ Molecules and the JM Region in the Tethered Kinase Simulations Normalized frequency of contacts between the JM and kinase domains and the PIP_2_-containing membrane for the main interaction mode (see Figures 5A and 5B). Red depicts interactions with PIP molecules, green with PS, and gray with PC. Orange bars indicate contacts between the JM and kinase domains. The inset illustrates contacts between the JM regions (Cα as orange spheres) and the surface of the kinase coded (white to orange) for the frequency of contacts to the JM. See also Figure S5 for the superimposition of the CG model onto the X-ray structure of the autoinhibited kinase EphB2. See also Figure S6 for a comparable analysis of the atomistic simulations of mode1 and mode2, and see Video S3.

The JM segment of a number of different RTKs plays a key role in modulating kinase function (Arkhipov et al., 2013, Jura et al., 2009, Lemmon and Schlessinger, 2010, Matsushita et al., 2013). We modeled the JM of EphA2 as a flexible (albeit within the known limitations of the CG forcefield) segment tethered at its N-terminus to the membrane by the TM domain and attached to the kinase at its C-terminus (see Figure 1B). We performed three repeat simulations each of duration 3 μs (see Table 1). In each simulation, the JM segment folds up within 0.2 μs, pulling the kinase domain onto the membrane (see Video S2). In each simulation, only interaction mode 1 is seen for the (wild-type) kinase PMI (see Figures 5A, 5B, and 6). Thus, the presence of the JM domain seems to bias the kinase toward binding in a “productive” mode with the activation loop exposed. This may have important consequences for the function of the kinase, as the JM segment may be seen as a scaffold to maintain the kinase domain in a configuration with the activation loop accessible for phosphorylation. The P1, P2, and P1+P2 mutations (see above) promote a shift to the “unproductive” mode 2 in which the activation loop is masked by the membrane (see Figure S4). We analyzed the interaction of the JM segment with the kinase domain. On folding against the membrane, the JM segment mainly interacted with the N-lobe of the kinase and near the activation loop (see Figure 6). In the 100-ns duration atomistic simulations (see above and Figure S6), both interaction modes 1 and 2 yield similar interactions between the JM segment and the kinase. A structure of the EphB2 kinase crystalized with a part of its JM segment in an autoinhibited form (PDB ID 1JPA) (Wybenga-Groot et al., 2001) may be compared with our model. Interestingly, we see the flexible JM region exploring conformations close to that in the autoinhibited structure of EphB2 (see Figure S5 and Video S3), although this should be interpreted cautiously given the simplifications inherent in the CG model (see below) employed in these simulations. Thus, we postulate that the flexible JM segment may explore a range of conformations on the surface of the N-lobe of the kinase.

**Table 1:** Summary of Simulations

Protein	Bilayer Composition	Particles or Atoms	Duration (μs)
**CG Simulations**			

Kinase WT^a^	PC:PS 70:30	30,240	10 × 3.0
Kinase WT^a^	PC:PS 60:40	33,100	10 × 3.0
Kinase WT^a^	PC:PS:PIP_2_ 90:5:5	33,070	10 × 3.0
Kinase mutant P1^a^	PC:PS:PIP_2_ 90:5:5	33,070	10 × 3.0
Kinase mutant P2^a^	PC:PS:PIP_2_ 90:5:5	33,070	10 × 3.0
Kinase mutant P1+P2^a^	PC:PS:PIP_2_ 90:5:5	33,060	10 × 3.0
Kinase WT^a^	PC:PS:PIP_3_ 83:15:2	33,080	10 × 3.0
TM + JM-small (20 residues)	PC:PS:PIP_2_ 90:5:5	82,375	1 × 3.0
TM + JM (40 residues)	PC:PS:PIP_2_ 90:5:5	135,280	1 × 3.0
TM + JM + kinase WT	PC:PS:PIP_2_ 90:5:5	164,018	3 × 3.0
TM + JM + kinase mutant P1	PC:PS:PIP_2_ 90:5:5	163,576	3 × 3.0
TM + JM + kinase mutant P2	PC:PS:PIP_2_ 90:5:5	163,963	3 × 3.0
TM + JM + kinase mutant P1+P2	PC:PS:PIP_2_ 90:5:5	164,003	3 × 3.0

**Atomistic Simulations**			

TM + JM + kinase WT mode1	PC:PS:PIP_2_ 90:5:5	863,343	0.1
TM + JM + kinase WT mode2	PC:PS:PIP_2_ 90:5:5	867,120	0.1

Summary of the simulations performed. CG, coarse-grained; WT, wild-type; JM, juxtamembranes; PC, phosphatidylcholine; PS, phosphatidylserine; PIP_2_, phosphatidyl inositol 4,5-bisphosphate; PIP_3_, phosphatidylinositol (3,4,5)-trisphosphate. See also Figures 1 and S1 for descriptions of the simulation systems.

a Simulations in which the kinases domain is not “tethered” via the TM and JM regions.

Video S2. Diffusion of the Tethered Kinase Domain toward a Model Membrane Containing PC, PS, and PIP_2_ Lipids, Related to Figures 5 and 6 The colored bar at the bottom shows the distance in between the center of masses of the kinase and the membrane from white (>12 nm) to black (∼4 nm).

Video S3. Folding of the JM Region (Orange) Seen in the Simulations Compared with the Crystal Structure of the Autoinhibited State of the EphB2 Kinase (PDBid: 1JPA; in Gray), Related to Figure 6 The conserved residues are displayed in licorice format for the EphB2 kinase and in van der Waals spheres format for the CG model.

### Recruitment of Anionic Lipids by the Cytosolic Domains of the EphA2 Receptor

**Figure 7:**
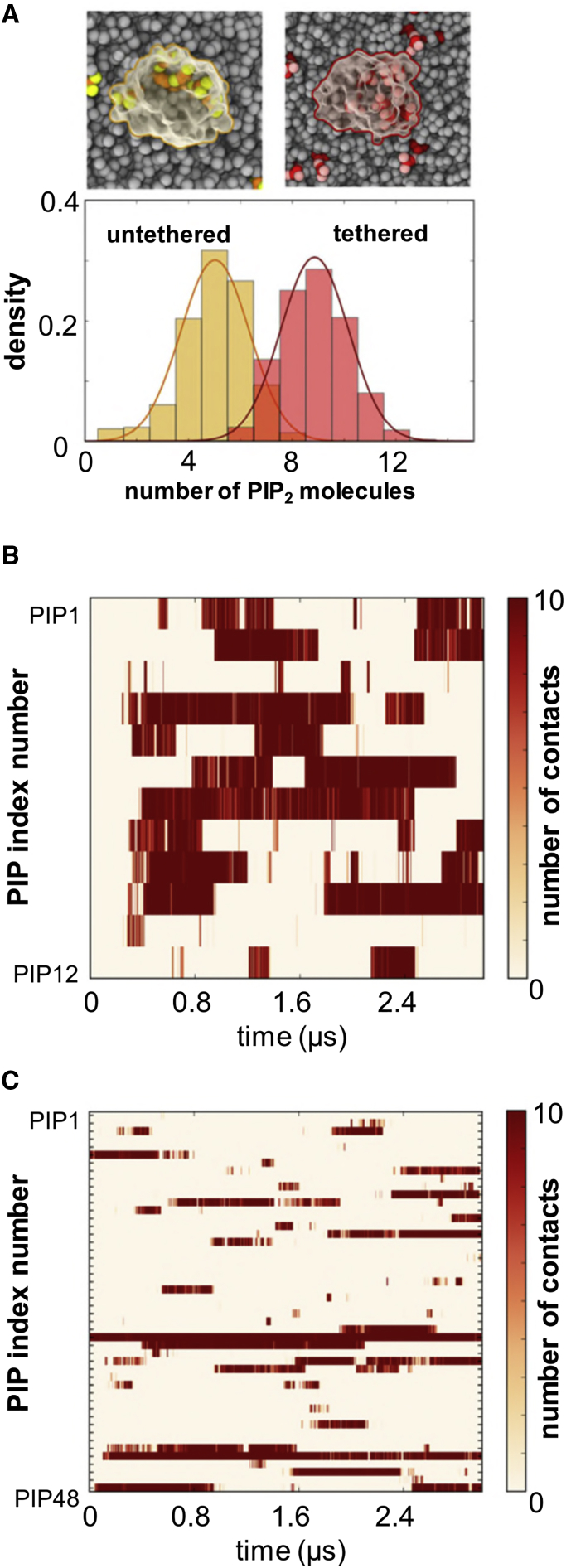
Nanoclustering of PIP_2_ Molecules by the JM + Kinase (A) Distribution of PIP_2_ molecules around the untethered (orange) and tethered (red) kinase bound to the membrane. (A PIP_2_ molecule is considered as interacting if its head group is at less than 0.8 nm from the protein.) The curves depict Gaussian density functions fitted to each distribution, with means of five and of nine PIP_2_ molecules for the untethered and tethered kinase, respectively. These are illustrated in the two insets above, with the headgroups of the PIP_2_ molecules shown as yellow or red spheres. See also Figure S7, which shows the slower diffusion of PIP_2_ in comparison with other lipids. (B and C) Time courses of PIP_2_ molecules interacting (i.e., within the 0.8-nm cutoff) with the untethered (B) or tethered (C) kinase domain. See also Figure S8 for analysis of JM segments interacting with the membrane.

We then investigated how interactions with the different domain regions of the cytosolic part of EphA2 may reorganize the local lipid environment around receptor. In these simulations we focused on PIP_2_, as this is the main PIP present in the plasma membrane. Modulation of the local lipid environment by membrane-bound proteins has been observed in a number of studies (e.g., Ni et al., 2017, van den Bogaart et al., 2011). The interaction of the kinase domain with the membrane resulted in local, i.e., nanoscale, clustering of PIP_2_ molecules in the bilayer around the bound protein domains (Figure 7). Comparing the simulations of the kinase alone and together with the JM segment showed some differences: more PIP_2_ molecules interacted with the protein in simulations with the JM segments than without (on average 5 PIP_2_ molecules compared with 9 PIP_2_ molecules, respectively; Figure 7A). In both cases, some PIP_2_ molecules form strong interactions persisting throughout the simulations. This can also be seen in the slower diffusion of PIP_2_ molecules in comparison with other lipids (see Figure S7). Other PIP_2_ molecules interact more transiently (see Figures 7B and 7C). Thus, PIP_2_ molecules form transient clusters around the kinase. Such interaction of the JM + kinase region leading to nanoclustering of PIP_2_ molecules in the vicinity of the receptor may aid the recruitment of further receptors, which in turn would be anticipated to facilitate autophosphorylation of kinase domains within a cluster (see below). Analysis of the interaction of the JM domain with the membrane showed substantial contributions from the N-terminal part of the segment (see Figure 6). To better assess the interaction of the JM with the membrane, we performed simulations with only the TM domain plus the extended/unfolded JM segment containing just the first 20 (JM-small) or all 40 residues (JM long) (see Figure 1B). In both cases we see substantive interactions with, and nanoclustering of, PIP_2_ (see Figure S8) by positively charged residues. The extent of this interaction with PIP_2_ molecules is reduced somewhat when the JM is attached to the kinase: some residues in the JM that interact with PIP_2_ in the absence of the kinase domain interact with the N-lobe of the kinase when the latter is included. Thus, there is a dynamic balance for the JM region between recruitment of PIP_2_ molecules and interaction with the kinase domain. Comparable clustering of PIP_2_ molecules has been seen in a number of other systems where basic residues interact with the cytoplasmic surface of the cell membrane, e.g., for syntaxin-1A (in both experiments and in CG simulations) (van den Bogaart et al., 2011), and for K-Ras4A (in atomistic simulations) (Li and Buck, 2017). Other examples include transmembrane receptors such as EGFRs (Abd Halim et al., 2015) and B cell receptors. For B cell receptors, it was shown that PIP_2_ binding modulates receptor activity and PIP_2_ production outside receptor microclusters through a positive signaling feedback loop (Xu et al., 2017).

We note that the TM + JM-small system construct is similar to those used in our earlier comparative study of lipid interactions with the JM + TM regions of all 58 human RTKs (Hedger et al., 2015). Given the conservation of JM/PIP_2_ interactions seen in the earlier study, and the enhanced clustering in the presence of the kinase domain seen in the current study (comparing Figures 7 and S8), this suggests that nanoclustering of PIP_2_ molecules is likely to be a general property of RTKs.

### Implications for Receptor Function

**Figure 8:**
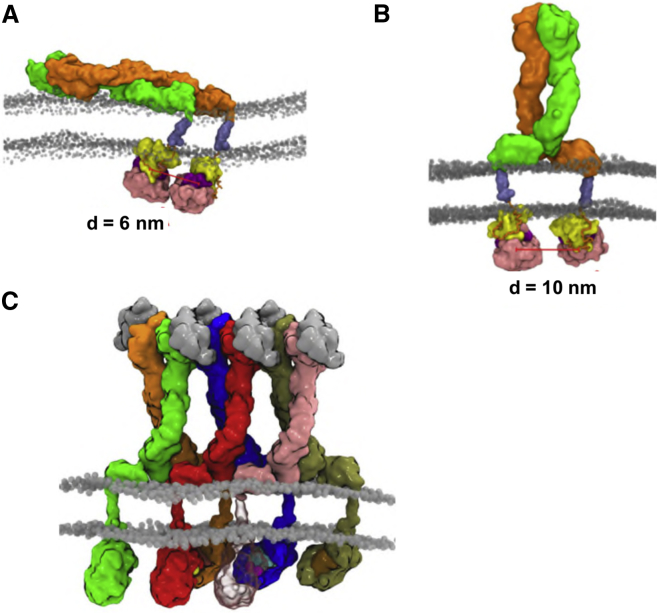
Integrative Model of the EphA2 Receptor at the Membrane (A) Unliganded EphA2 dimer with the two ectodomains (shown in green and orange) on the extracellular surface the membrane, such that the two TM domains are apart and the center of mass distance between the two kinases is ca. 6 nm. (B) Liganded conformation of the EphA2 dimer for which the center of mass distance between the two kinases is ca. 10 nm. (C) An illustrative example of a higher order receptor cluster the formation of which is triggered by ligand binding. The ectodomain array was derived from (Seiradake et al., 2013), the position of the FN2 at the membrane surface by combined MD simulations and biophysical assays (Chavent et al., 2016), and the position of the kinase domain was extracted from CG simulations for mode 1 (see Figure 3).

We have integrated the results of the simulations of the TM + JM + kinase we report here with our previous models of the ecto + TM domains of the EphA2 receptor (Chavent et al., 2016). We superimposed our simulation trajectories for the kinase domain onto our previous models for an interacting pair of ectodomains (in either the unliganded, or liganded state) using the TM domain as the common reference frame. We then analyzed the distance between the centers of mass of the two kinase domains in each of the two composite models. This analysis reveals that the kinase domains may adopt different relative orientations as a function of the interplay between the conformations of the ectodomain and of the TM domain (Figure 8). In the unliganded conformation, for which our previous work suggests the ectodomain dimer lies parallel on the membrane (Chavent et al., 2016), the two kinase domains are separated by ca. 6 nm (Figure 8A), which is likely to be too far apart to permit autophosphorylation. Similarly, if the kinases are positioned according to the liganded dimer arrangement of ectodomains, which our previous analysis positioned “upright” relative to the membrane, the kinases of the dimer are separated by ca. 10 nm (Figure 8B), again too far apart to allow for autophosphorylation. Taken together, this analysis suggests autophosphorylation may optimally require interactions between adjacent dimers within a higher order receptor cluster (see Figure 8C for an illustrative example of such a receptor cluster), the formation of which is triggered by ligand binding (Seiradake et al., 2010). Autophosphorylation between adjacent clustered dimers has recently been suggested for, e.g., the EGFR (Huang et al., 2016, Needham et al., 2016). Such a model may underlie the importance in signaling of clustering of EphA2 receptors via a seeding mechanism (Seiradake et al., 2010). These models may also help us to more fully understand the role of the C-terminal SAM domain on receptor oligomerization and activation (Shi et al., 2017, Singh et al., 2017).

### Methodological Reflections

This study has largely employed CG simulations, employing the MARTINI forcefield (Monticelli et al., 2008). This method has been used extensively to explore interactions of both integral (Arnarez et al., 2013, Hedger et al., 2016b, Periole et al., 2012) and of peripheral (Kalli and Sansom, 2014, Kalli et al., 2010, Naughton et al., 2016) membrane proteins with lipid bilayers, and has yielded results that agree well with available experimental data. However, it is useful to reflect upon the possible limitations of the approach, especially in the context of studies of both integral (Javanainen et al., 2017) and peripheral (Herzog et al., 2016, Li and Gorfe, 2013) membrane proteins, which suggest that the forcefield may be too “sticky,” i.e., that protein/protein and protein/lipid interactions may be too strong leading to aggregation in simulations.

Our experience via comparison with a range of experimental data is that the MARTINI forcefield provides reasonable agreement with experimental data for protein interactions within and with membranes. For example, CG estimations of the free energy landscape for dimerization of the glycophorin A TM domain, which has been the subject of intense scrutiny, yield a predicted dissociation constant in agreement with experimental estimates (Domański et al., 2017). For peripheral membrane proteins, we have focused on another experimentally well characterized system, namely PH domains. CG simulations have revealed that both the structural (Yamamoto et al., 2016) and energetic aspects (Naughton et al., 2016, Naughton et al., 2018) of PH domain/lipid bilayer interactions are in agreement with experimental estimates. Indeed, comparison of predicted free energy landscapes of interaction with experimental protein/lipid dissociation constants for PH domains suggested that the predicted free energies of interaction were if anything too small unless one assumed that a PH domain interacted with more than a single PIP molecule within a membrane (Naughton et al., 2016, Naughton et al., 2018). Thus overall, the MARTINI forcefield seems to give a reasonable representation of protein/membrane interactions, although doubtless further theoretical and experimental studies would refine the degree of quantitative agreement for a wider range of systems.

One aspect of protein/lipid interactions for which the MARTINI forcefield is limited is prediction of selectivity between PIP_2_ and PIP_3_ binding to peripheral membrane proteins (Naughton et al., 2016, Naughton et al., 2018). This is perhaps not surprising given the approximations of the CG forcefield, but it is relevant to discussions of the kinase and JM domain interactions of the EphA2 receptor (see above). It is also likely that the approximations used to treat electrostatic interactions within the MARTINI model are such that our CG simulations will be unable to accurately reflect effects of ionic strength on EphA2 interactions with PIPs. Taken together, these approximations may have effects on the prediction of, e.g., PIP_2_ versus PIP_3_ selectivity. It is likely that more extensive all atom simulations (Li and Buck, 2017) may be required to resolve this in the future.

### Conclusion

We have demonstrated the contribution of PIP_2_-mediated interactions between the JM + kinase domains and the membrane in determining the overall configuration of the EphA2 receptor within cell membranes. The JM region is flexible and can interact with anionic lipid headgroups in the bilayer and/or the surface of the kinase domain. The kinase domain can also interact directly with these lipid headgroups. Interaction of the JM + kinase region leads to nanoclustering of PIP_2_ molecules in the vicinity of the receptor. This may aid the recruitment of further receptors, either singly or as dimer, in turn facilitating autophosphorylation of kinase domains of adjacent dimers within a cluster.

The proposed interaction of an Eph receptor JM + kinase region with PIPs is a novel aspect of receptor function, whose physiological relevance will need to be defined in future work. This proposal aligns with a growing list of receptors that respond functionally to PIP_2_ or PIP_3_ concentrations, often involving signaling feedback loops (e.g., EGFR [Toth et al., 2016] and B cell receptors [Wang et al., 2017]). Intriguingly, ours is not the first study to suggest cross-talk between Eph and PIP_2_ signaling pathways, suggesting that such feedback loops may exist also for Ephs. For example, class B Eph receptor activity was shown to increase the amount of PIP_2_ in membranes to regulate synaptic endocytosis (Irie et al., 2005). Furthermore, PI3K activity has been functionally linked to Eph signaling in a number of studies, particular in the context of disease (e.g., Jiang et al., 2015, Menges and McCance, 2008).

## STAR★Methods

### Key Resources Table

**Table:** 

REAGENT or RESOURCE	SOURCE	IDENTIFIER
**Chemicals, Peptides, and Recombinant Proteins**		

Human EphA2 kinase domain	N/A - this paper	
SF900II Media	Gibco	Cat#10902-088
Penicillin-Streptomycin	Sigma	Cat#P0781
Fugene transfection reagent	Promega	Cat#E2691
POPC	Avanti Polar Lipids	Cat#850457C
POPS	Avanti Polar Lipids	Cat#840034C
PIP_2_	Echelon Biosciences	Cat#P-4516
PIP_3_	Echelon Biosciences	Cat#P-3916
0.1μm Polycarbonate membrane	Whatman	Cat#800309

**Deposited Data**		

Coordinates of the models generated	N/A – this paper	https://zenodo.org/record/1228176#.Wt870mbpN25

**Experimental Models: Cell Lines**		

SF9 cells	ThermoFisher Scientific	Cat#11496015

### Contact for Reagent and Resource Sharing

Further information and requests for resources and reagents should be directed to and will be fulfilled by the Lead Contact, Mark Sansom (mark.sansom@bioch.ox.ac.uk).

### Experimental Model and Subject Details

For protein expression, Sf9 cells were cultured in suspension in SF900II media supplemented with 100μg/ml penicillin and 100μg/ml streptomycin to a density of 1.5×10^6^ cells/ml, at a temperature of 26-28°C.

### Method Details

#### CG-MD Simulations

Coarse-grained MD (CG-MD) simulations were performed using GROMACS 5.1 (www.gromacs.org) (Hess et al., 2008, Pronk et al., 2013) with the MARTINI 2.1 forcefield (Monticelli et al., 2008). To model the kinase domain, we used PDB structure 1MQB (Nowakowski et al., 2002). We removed the ligand and used Modeller (Fiser and Sali, 2003, Sali and Blundell, 1993) to add back missing loops and also to model the JM segment. To maintain the secondary structure of the protein in the CG simulations we applied elastic restraints within the kinase domain and within the TM domain, using a 0.7 nm cutoff (Periole et al., 2009). Elastic network restraints were not applied to the JM region (residues 22 to 57) which thus remained flexible. For systems with isolated kinase protein diffusing towards the membrane (see Table 1), we performed 10 simulations on different preassembled membranes: (i) a mixture of PC and PS (phosphatidyl choline and phosphatidyl serine, more fully POPC = 1-palmitoyl-2-oleoyl PC 1-palmitoyl-2-oleoyl PS respectively); (ii) a mixture of PC:PS:PIP_2_; and (iii) a mixture of PC:PS:PIP_3_. These ratios were selected to approximate a mammalian plasma membrane (inner leaflet) and to maintain the overall lipid headgroup charge as for the 70:30 PC:PS system. For the larger systems containing the TM domain and different extension of the JM and the kinase (see Table 1), we used a preassembled membrane with a 90:5:5 PC:PS:PIP_2_ ratio. Further simulation details can be found in Table 1. Water and counterions (Na^+^ and Cl^-^) were added to equilibrate the system. After 100 steps of steepest descent, we performed 5 nanoseconds of equilibration (during which the protein was restrained) before the production runs. The temperature was 323K. Electrostatic interactions were shifted to zero between 0 and 1.2 nm and the Lenard-Jones interactions between 0.9 and 1.2 nm. A Berendsen thermostat in combination with a Berendsen barostat (Berendsen et al., 1984) with a coupling constant of 1.0 ps, a compressibility of 5.0 x 10^-6^ bar^-1^, and a reference pressure of 1 bar were used, and the integration timestep was 20 fs.

#### Atomistic Simulations

The TM+JM+kinase CG-systems (protein and lipids) were converted to atomistic resolution using the CG2AT protocol (Stansfeld and Sansom, 2011). Atomistic simulations were performed using the GROMOS96 53a6 force field. Water and ∼150 mM NaCl were added. 5000 steps of steepest descent minimization were followed by a 5 ns equilibration simulation during which position restraints on the protein were gradually removed. The equilibrated system was then subjected to a 100 ns unrestrained MD simulation. Electrostatics were modeled using the particle mesh Ewald procedure (Darden et al., 1993). All bonds were constrained with the P-LINCS algorithm (Hess, 2007) The simulation was performed at constant temperature (310 K), pressure, and particle number using semi-isotropic pressure coupling with the Parrinello-Rahman barostat (Parrinello and Rahman, 1981) and the V-rescale thermostat (Bussi et al., 2007). The integration time step was 2 fs.

#### Simulation Analysis

VMD (Humphrey et al., 1996) was used to visualize structures and was combined with Tcl scripts to analyse the simulations. The rotation versus distance matrix analyses were performed as described in (Yamamoto et al., 2016). Thus we calculated the 2-dimensional normalized histogram of *R*_*zz*_ and *d*_*z*_, where *d*_*z*_ is the perpendicular distance between the centres of mass of the protein domain and the lipid membrane, and where *R*_*zz*_ is the *zz* component of the rotational matrix required for least squares fitting of a orientation onto a reference orientation. *R*_*zz*_ was calculated by using the *g_rotmat* command in GROMACS (www.gromacs.org) (Hess et al., 2008, Pronk et al., 2013). The value of *R*_*zz*_ in the density map varies depending on the reference orientation of the PH domain relative to the membrane. The change in the normalized density map of system can be calculated from *ΔD(R*_*zz*_*, d*_*z*_*) = ρ(R*_*zz*_*, d*_*z*_*) / ρ*_*0*_, where *ρ(R*_*zz*_*, d*_*z*_*)* and *ρ*_*0*_ are probabilities at a bin *(R*_*zz*_*, d*_*z*_*)* and a reference point (which corresponds to the global maximum), respectively.

#### Cloning and Protein Purification

A construct of human EphA2 kinase domain (residues 595-897, Uniprot: P29317) was cloned into pBacPAK9 vector (Clontech) using EcoRI and XhoI sites using standard cloning techniques. Sf9 cells cultured in SF900II media (Invitrogen) supplemented with 100μg/ml penicillin and 100μg/ml streptomycin, were co-transfected with recombinant transfer vector containing EphA2 kinase domain and linearized viral DNA using Fugene (Promega). Supernatant containing the recombinant baculovirus were harvested 5 days post transfection. Viral stocks were amplified to obtain P2 stock. For protein expression, SF9 cells were cultured in suspension in SF900II media supplemented with 100μg/ml penicillin and 100μg/ml streptomycin to a density of 1.5×10^6^ cells/ml. 400ml cells were infected with 10ml of P2 viral stock. Infected cells were harvested after 72 hours of incubation at 27°C and 120 RPM. Protein purification was carried out using standard Ni-affinity and size exclusion chromatography.

#### Liposome Pull-Down Assay

Liposomes were prepared by drying 1-palmitoyl-2-oleoyl-sn-glycero-3-phosphocholine (POPC) (Avanti Polar lipids), 1-palmitoyl-2-oleoyl-sn-glycero-3-phospho-L-serine (POPS) (Avanti Polar lipids), phosphatidylinositol 4,5-bisphosphate diC16 (PIP_2_) (Echelon Biosciences) and phosphatidylinositol 3,4,5-trisphosphate diC16 (PIP_3_) (Echelon Biosciences) in the desired ratios (w/w) overnight under vacuum. The lipid films were re-suspended in buffer (20mM HEPES, pH 7.4, 100mM NaCl) and subjected to 7 cycles of freeze-thaw using liquid nitrogen to generate liposomes. Liposomes were then extruded by passing them through a 0.1μm Polycarbonate membrane (Whatman). Final lipid concentrations were 2mg/ml. 100μl of liposomes were mixed with 50μl of EphA2 kinase domain protein (0.1mg/ml) and incubated at room temperature for 1 hour. Liposome-protein mixtures were centrifuged at 150,000×g for 30minutes at 20°C. Pellets were washed vigorously with buffer (20mM HEPES, pH 7.4, 100mM NaCl) and centrifuged again. These experiments were repeated 5 times and for each set the bound protein fractions was analysed by SDS-PAGE. The protein bands on gels were quantified by densitometry using Image Lab software. Averages of the ratio of intensities (bound: unbound) and SEM from all experiments were then calculated.

### Quantification and Statistical Analysis

In the liposome pull-down assay the experiments were repeated *n =* 5 times as specified in the figure legend. Averages of the ratio of intensities (bound: unbound) and SEM were calculated.

### Data and Software Availability

Coordinates of the models generated by this study (as final frames of atomistic simulations revealing the interactions of the transmembrane, juxtamembrane (JM), and kinase domains with the membrane) are available at: https://zenodo.org/record/1228176#.Wt870mbpN25.

## Supplemental Information

Document S1. Figures S1–S8

Document S2. Article plus Supplemental Information
