# Supplementary material for: Interactions of the EphA2 Kinase Domain with PIPs in Membranes: Implications for Receptor Function
Source: Structure. 2018 Jul 3;26(7):1025–1034.e2. doi: 10.1016/j.str.2018.05.003 (PMC6039763; doi:10.1016/j.str.2018.05.003)
Supplement: Document S1. Figures S1–S8 [file mmc1.pdf]

Structure, Volume *26*

Supplemental Information

Interactions of the EphA2 Kinase Domain with PIPs in Membranes: Implications for Receptor Function

Matthieu Chavent, Dimple Karia, Antreas C. Kalli, Jan Domański, Anna L. Duncan, George Hedger, Phillip J. Stansfeld, Elena Seiradake, E. Yvonne Jones, and Mark S.P. Sansom

SI Figures

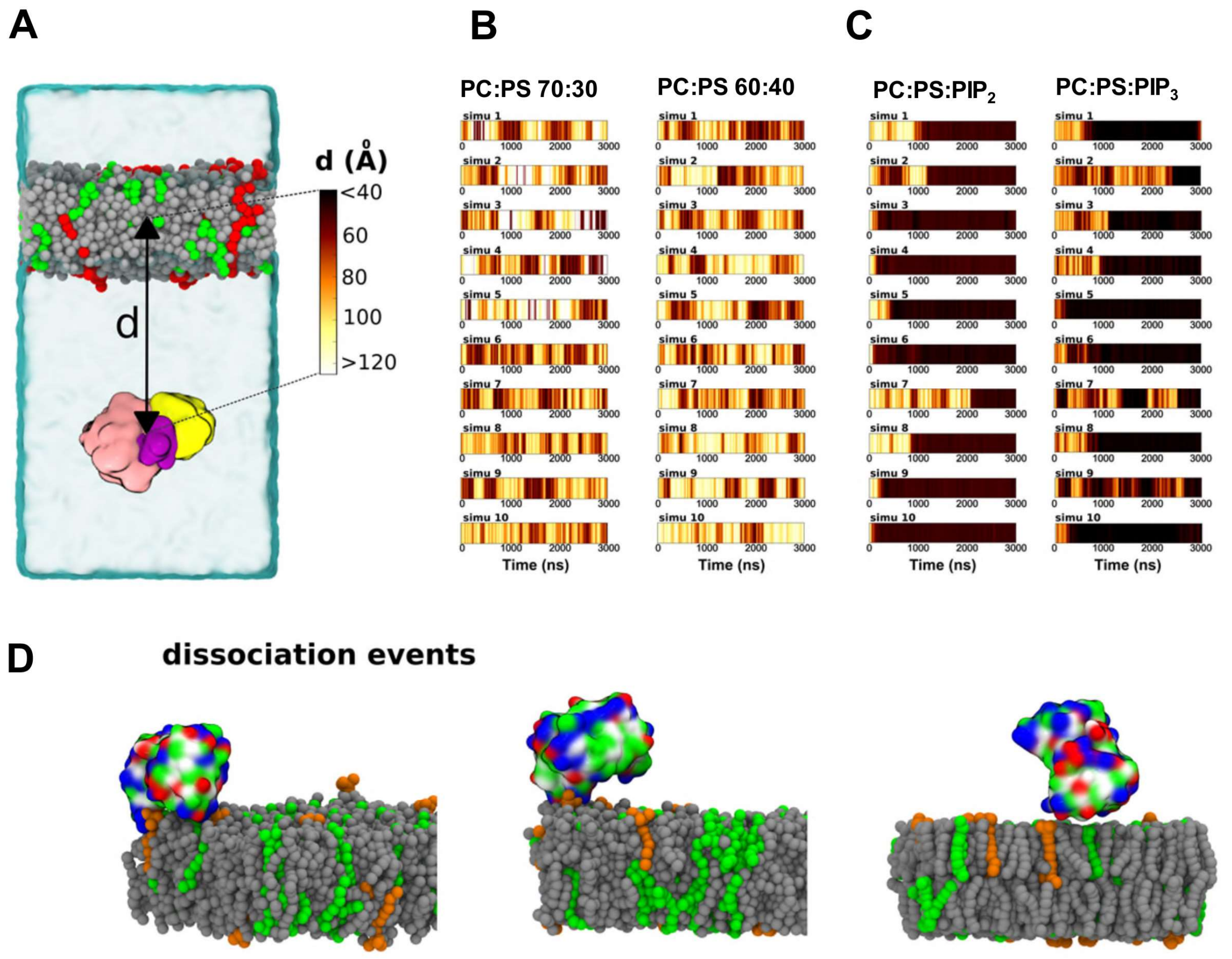

<u>*SI Figure S1, Related to Figure 1:*</u>

A Starting CG model of the (un-tethered) kinase domain adjacent to a membrane. The kinase is positioned 12 nm away from the membrane, allowing it to diffuse and rotate freely and to interact with the membrane (see Movie S1). The distance (*d*) between the centres of mass of the kinase and the membrane is monitored during the simulations.

B,C Evolution of the distance between the centres of mass of the untethered kinase domain and the membrane for simulations with different bilayer lipid compositions (see Table 1 for details). The distance between the centres of mass of the kinase and the membrane is depicted as a colour gradient from dark (short distance) to light (long distance). This gradient is then used to easily displayed stability of the membrane-kinase interaction. **D** Snapshots from a simulation of the interaction of the kinase domain with a PC:PS:PIP_3 bilayer (see Table 1 for details) in which an associated kinase domain subsequently dissociates from the bilayer.

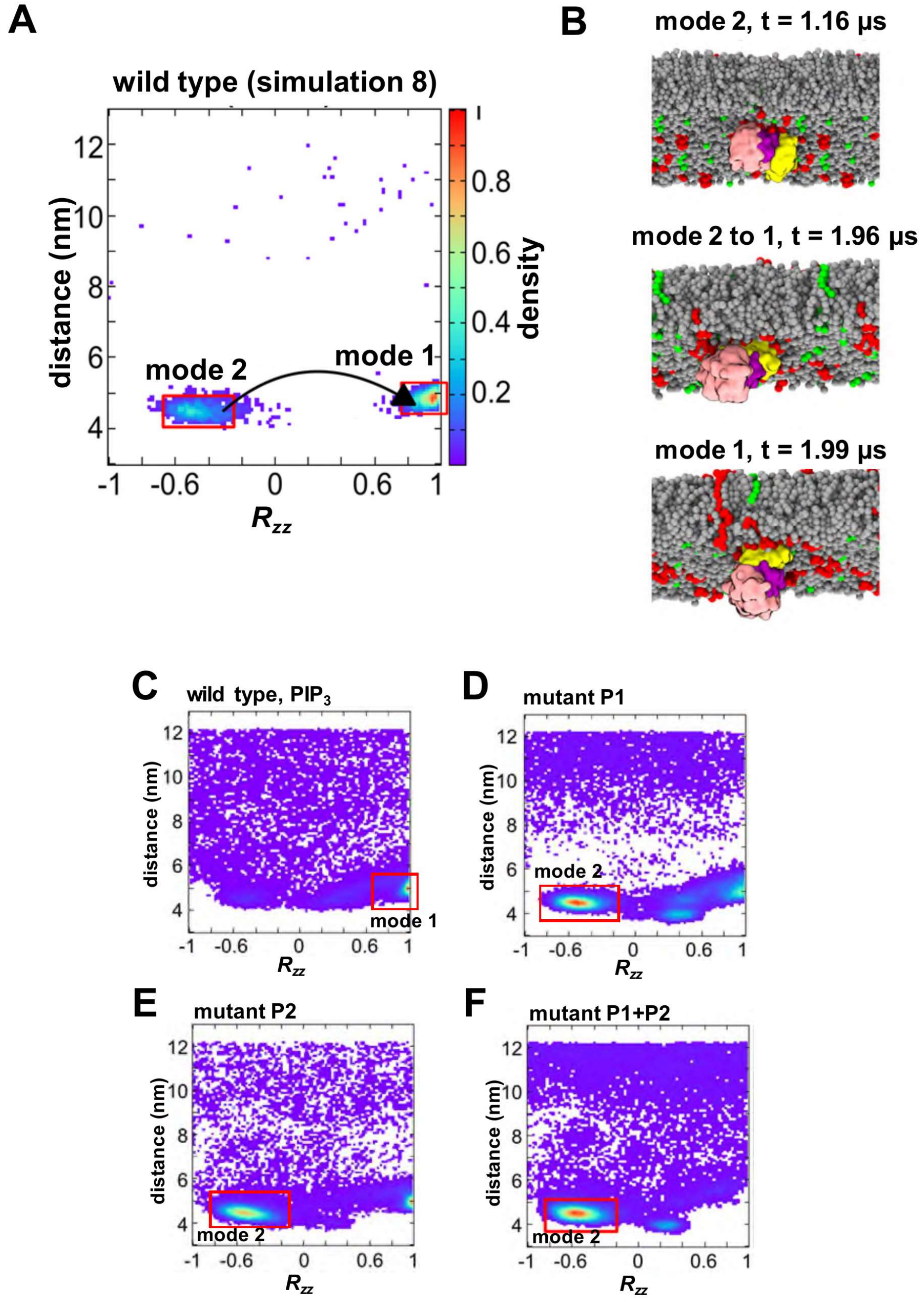

<u>*SI Figure S2, Related to Figure 3:*</u>

A Position and orientation of the untethered wild type kinase domain throughout a simulation during the transition from a mode 2 to a mode 1 interaction in the presence of a PC:PS:PIP_2 bilayer (see Table 1) displayed as a normalized density map showing the domain-bilayer centres-of-mass separation d and the R_{zz} component of the rotation matrix. The two main modes of interaction of the kinase with the bilayer are highlighted via red boxes.

B Snapshots from the simulation illustrating the transition from a Mode 2 to a Mode 1 interaction with the membrane. **C-F** Position and orientation of **C** the untethered wild type kinase domain with a PC:PS:PIP_3 bilayer and of **D,E,F** *in silico* mutant kinase domains with a PC:PS:PIP_2 bilayer, in each case derived from 10 simulations (see Table 1). The two main modes of interaction of the kinase with the bilayer are highlighted via red boxes. (The results of comparable analysis for the wild type simulation with PIP_2 interaction are presented in Figure 3 in the main manuscript.)

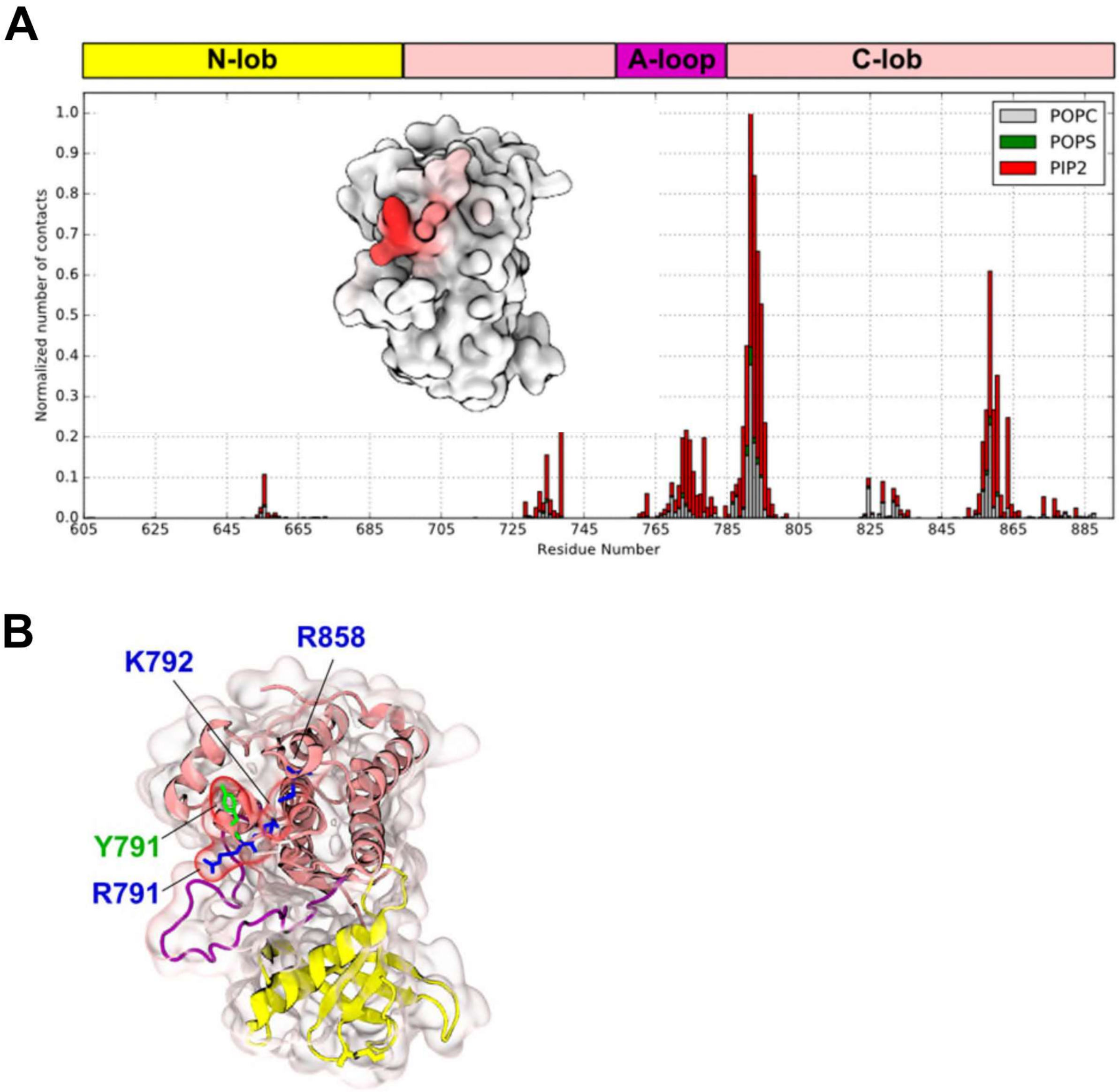

<u>SI Figure S3, Related to Figure 4:</u>

A Normalized frequency of contacts (defined as the relative number of interacting particles within a 0.8 nm cutoff distance of the PIP_2 headgroup) between the kinase domain and the PIP-containing membrane for the Mode 2 interaction. Red depicts interactions with PIP molecules, green with PS, and grey with PC. The interacting residues are all located in the C-terminal lobe of the kinase, as illustrated in the inset which depicts the contacts highlighted at the protein surface by a white to red gradient. **B** Residues (basic in blue, others in green) interacting with lipid molecules in the membrane for the Mode 1 interaction. (The results of comparable analysis for the Mode 1 interaction are presented in Figure 4 in the main manuscript.)

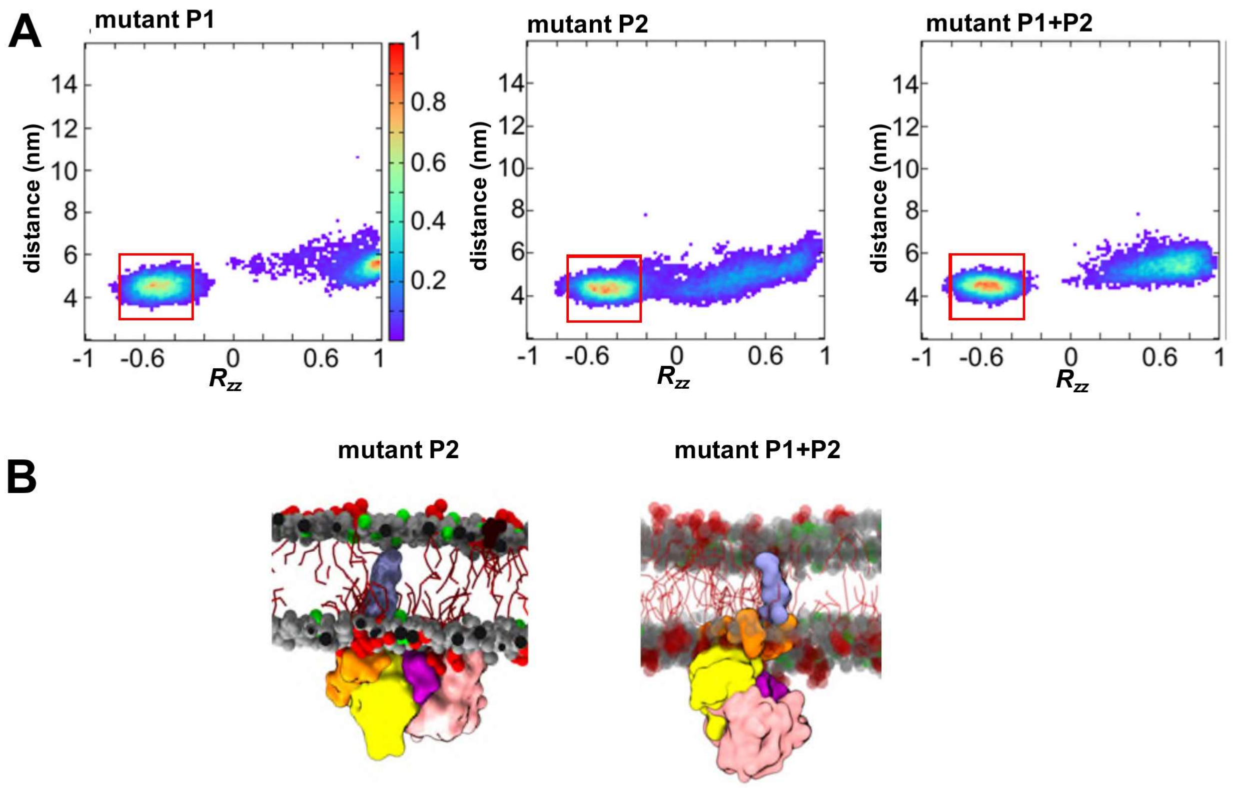

<u>*SI Figure S4, Related to Figure 5:*</u>

A Position and orientation of the tethered mutant kinase domains (derived from data acquired across the 3 simulations; see Table 1 for details) displayed as a normalized density map showing the domain-bilayer centres-of-mass separation *d* and zz component of the rotation matrix R_{zz}. The mode 2 interactions of the kinase with the bilayer are highlighted via a red box. **B** Examples of the mode 2 of interaction of the P2 and P1+P2 mutant kinase domains with the membrane. (See also the main text Figure 5 for comparable analysis of the wild type simulations.)

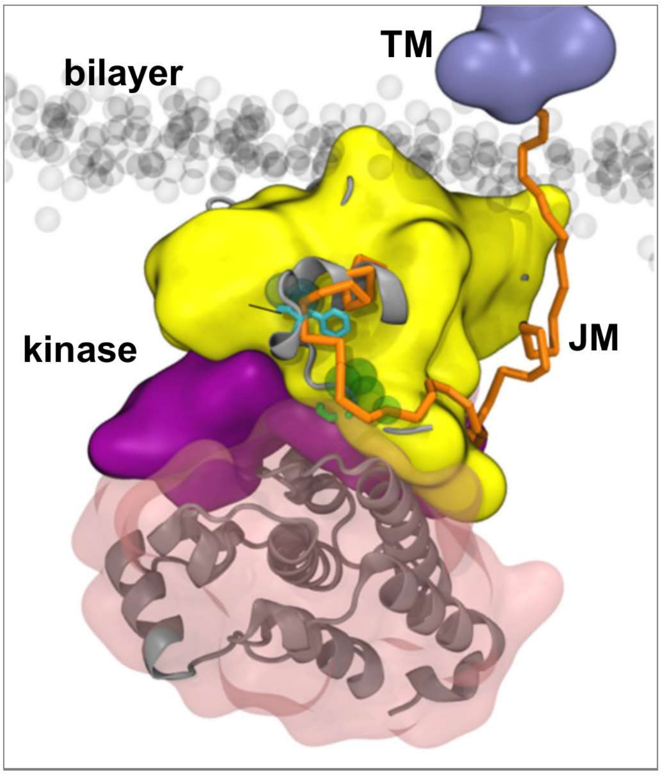

<u>*SI Figure S5, Related to Figure 6:*</u>

Comparison of the folding of the JM region (orange) seen in the simulations with the crystal structure of the autoinhibited EphB2 kinase (PDBid: 1JPA; in grey). The conserved residues are displayed in licorice format for the EphB2 kinase and in van der Waals spheres format for the CG model (also see movie S3).

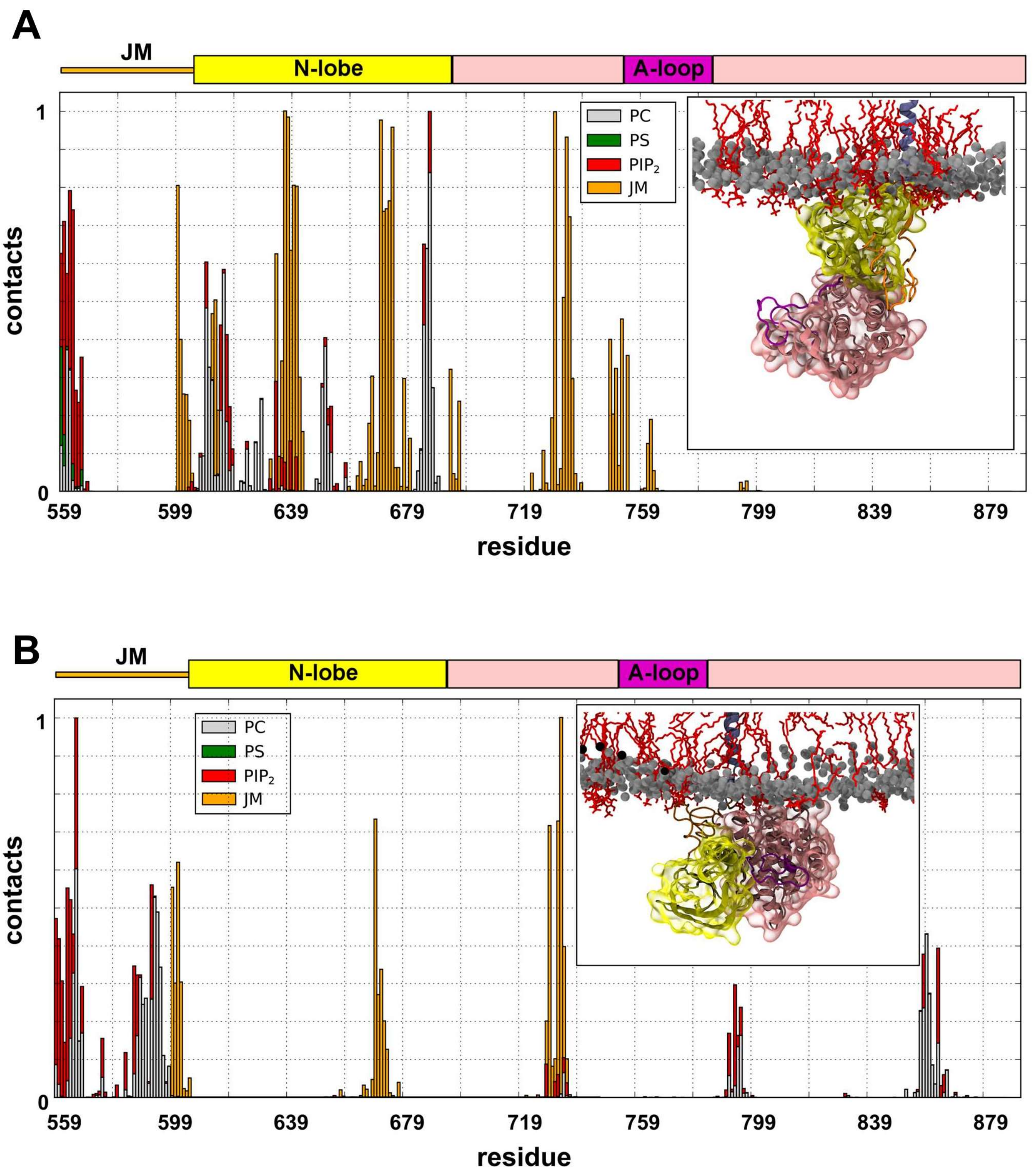

<u>*SI Figure S6, , Related to Figure 6.*</u>

Refining the two modes of interaction via short (100 ns) atomistic simulations. Normalized frequency of contacts between the JM and kinase domains and the PIP_2-containing membrane are shown, derived from the atomistic simulations for interaction mode1 (**A**) mode2 (**B**). Red depicts interactions with PIP molecules, green with PS, and grey with PC. Orange bars indicate contacts between the JM and kinase domains. The inset illustrates the position of the kinase and JM domains at the membrane surface (with lipid phosphate atoms in grey and PIP_2 molecules in red).

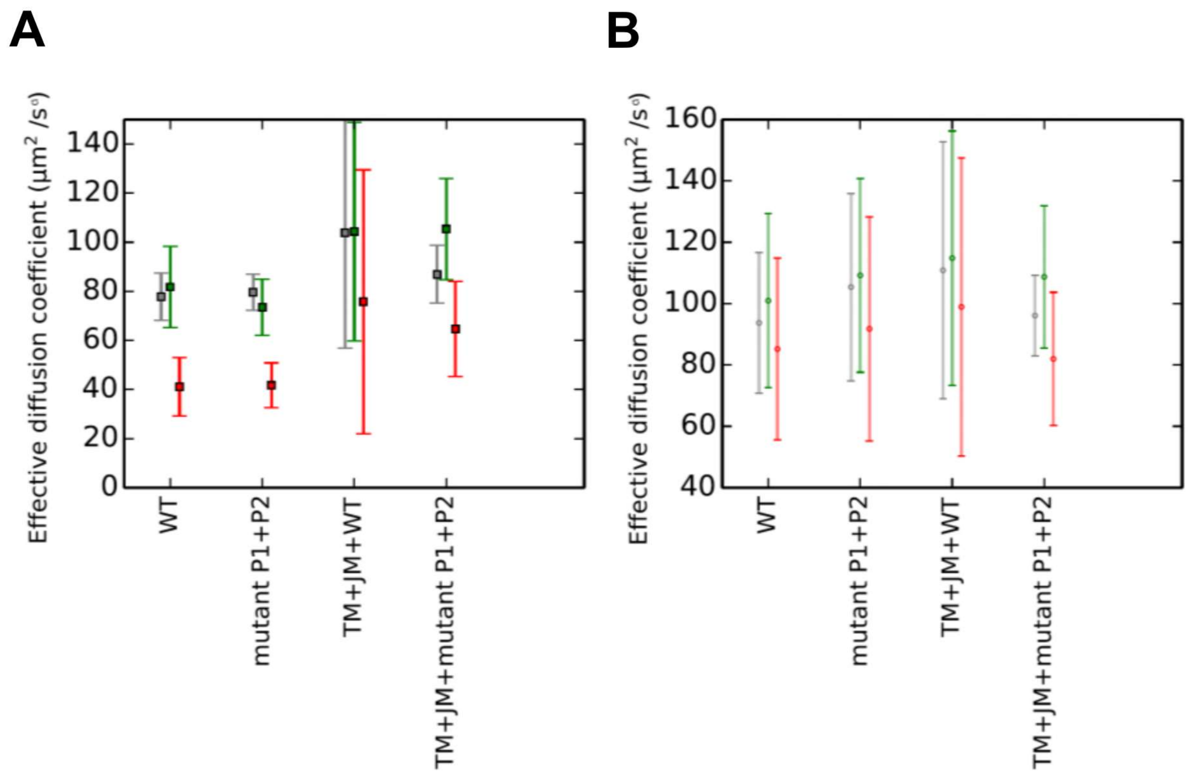

<u>*SI Figure S7, Related to Figure 7:*</u>

Effective diffusion coefficients of PIP molecules relative to other lipids (PS and PC) from both the untethered simulations (Kinase WT and mutant P1+P2) and from the TM+JM+ kinase WT and TM+JM+ kinase mutant P1+P2 simulations in bilayers containing PIP_2; see Table 1 for details). Diffusion coefficients for PIP_2 are in red, other lipids are in green (PS) and grey (PC). Lipids from the two leaflets in each ensemble of simulations are shown separately: on the left panel (**A**) are lipids from the leaflet interacting with the protein; on the right panel (**B**) are lipids in the opposite leaflet. Error bars show standard deviation diffusion coefficients. Diffusion analysis was carried out on trajectories from which the center of mass motion of the protein had been removed. Only the part of the trajectory where the protein had a stable interaction with the membrane was used, that is the 50-point running average protein-membrane distance was below 5.5 nm until the end of the trajectory. Time- and ensemble-average mean squared displacement (MSD) for each lipid species was extracted using a time step of 0.4 ns. Effective diffusion coefficients (D_α) were obtained by fitting the equation $MSD = 4D_\alpha \Delta t^\alpha$ to graphs of MSD *vs* Δt, with Δt in the range 0.4 ns to 100 ns. Error bars show the standard deviation of the ten simulations performed for each category. Scripts to perform this analysis made use of MDAnalysis and https://github.com/tylerjereddy/diffusion_analysis_MD_simulations.

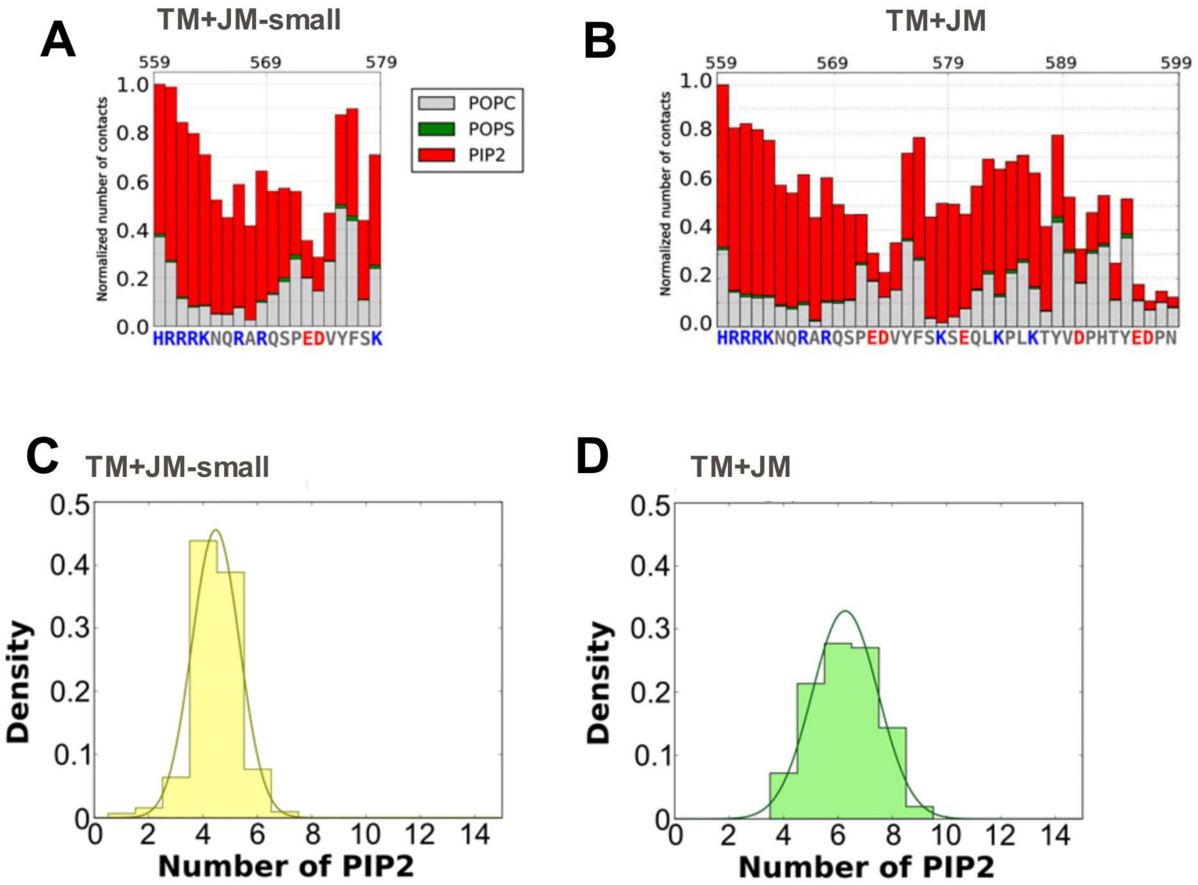

<u>*SI Figure S8, Related to Figure 7:*</u>

A,B Normalized frequency of contacts (defined as the relative number of interacting particles within a 0.8 nm cutoff distance of the PIP_2 headgroup) between the JM region and the PIP-containing membrane for the **A** TM+JM-small and **B** TM+JM simulations (see Table 1 for details) Red depicts interactions with PIP molecules, green with PS, and grey with PC. **C,D** Distribution of PIP_2 molecules around the **C** JM-small, **D** JM (40 residue) in the corresponding simulations (see Table 1 for details). A PIP_2 molecule is considered as interacting if its headgroup is at less than 0.8 nm from the protein. The curves depict Gaussian density functions fitted to each distribution. (See Figure 7A in the main text for comparable analysis of the untethered and tethered kinase simulations).
